# Synthesis and evaluation of anti-oxidant and cytotoxic activities of novel 10-undecenoic acid methyl ester based lipoconjugates of phenolic acids

**DOI:** 10.3762/bjoc.13.4

**Published:** 2017-01-04

**Authors:** Naganna Narra, Shiva Shanker Kaki, Rachapudi Badari Narayana Prasad, Sunil Misra, Koude Dhevendar, Venkateshwarlu Kontham, Padmaja V Korlipara

**Affiliations:** 1 Centre for Lipid Research, CSIR-Indian Institute of Chemical Technology, Uppal Road, Hyderabad 500007, India; 2 Academy of Scientific and Innovative Research, New Delhi, India; 3 Biology Division, CSIR-Indian Institute of Chemical Technology, Uppal Road, Hyderabad 500007, India

**Keywords:** anticancer, anti-oxidants, caffeic acid, coumaric acid, ferulic acid, phenolic lipids, sinapic acid, undecenoic acid

## Abstract

The synthesis of five novel methyl 10-undecenoate-based lipoconjugates of phenolic acids from undecenoic acid was carried out. Undecenoic acid was methylated to methyl 10-undecenoate which was subjected to a thiol–ene reaction with cysteamine hydrochloride. Further amidation of the amine was carried out with different phenolic acids such as caffeic, ferulic, sinapic, coumaric and cinnamic acid. All synthesized compounds were fully characterized and their structures were conﬁrmed by spectral data. The anti-oxidant activity of the synthesized lipoconjugates of phenolic acids was studied by the 2,2-diphenyl-1-picrylhydrazyl (DPPH) radical scavenging assay and also by the inhibition of linoleic acid oxidation in micellar medium by differential scanning calorimetry (DSC). The prepared compounds were also screened for their cytotoxic activity against five cell lines. It was observed that the lipoconjugates of caffeic acid, sinapic acid, ferulic acid, and coumaric acid displayed anticancer and anti-oxidant properties. The anticancer properties of these derivatives have been assessed by their IC_50_ inhibitory values in the proliferation of MDA-MB231, SKOV3, MCF7, DU 145 and HepG2 cancer cell lines.

## Introduction

Phenolic compounds are a class of natural compounds which are found ubiquitously in the plant kingdom. They are reported to possess a wide range of biological properties like anti-oxidant, antimicrobial, anti-inflammatory, anticarcinogenic and antiviral activities [1]. The phenolic acids are also reported to show in vitro anti-oxidant activity against many reactive oxygen species and to protect neuronal cells against various types of oxidative damage [2–3]. To increase the effectiveness of phenolic compounds, their lipophilization has been the choice of derivatization as it provides beneficial effects of both the phenolics and the lipid involved in one chemical entity [4–5]. Lipids, especially fatty acids and their derivatives are known for their broad spectrum of activity which expands their application in developing new hybrid biomolecules which help in host defenses against potential pathogenic microbes. Research interest in producing new phenolipids has been increasing due to the potential applications of such products in biomedical fields. Earlier reports on the production of phenolipids were focused on the incorporation of phenolic compounds into triglycerides where a number of phenolic acids were transesterified with different oils or triglycerides [6]. Apart from these structured phenolipids, different fatty acids were esterified with phenolic compounds to produce novel esters which were evaluated for anti-oxidant and antimicrobial activities [7–8]. However, there are very few reports where the phenolic acids have been derivatized with other functionalities apart from esters. The reported compounds other than esters were amides where bioconjugates of fatty acids and amino acids were prepared and evaluated for their anti-oxidant activity by a DPPH radical assay [9]. In view of developing new conjugates of phenolic lipids, we have synthesized novel derivatives of phenolic lipids from undecenoic acid where the phenolic acids were linked to the olefinic group of undecenoic acid via a thioamide spacer. Among the various fatty acids reported, 10-undecenoic acid is unique due to its bifunctional nature with an odd-numbered carbon atom chain length derived from castor oil. There have been several reports on the synthesis and evaluation of undecenoic acid-based derivatives due to its wide applicability ranging from biological activity, natural products and polymer applications [10–11]. This type of compounds could be useful as potential novel lipid derivatives because of the presence of lipophilic chain and the phenolic amide conjugate.

## Results and Discussion

### Synthesis

**Scheme 1:**
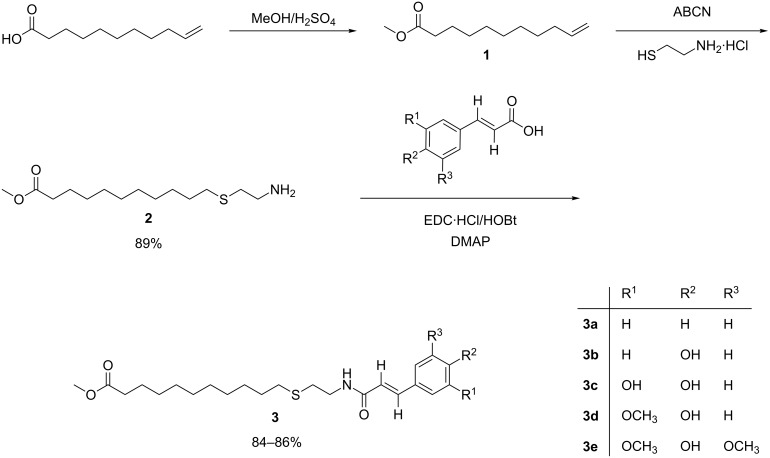
Synthetic procedure for the preparation of 10-undecenoic acid methyl ester-based lipoconjugates of phenolic acids.

10-Undecenoic acid was chosen as the lipid part as the derivatives of undecenoic acid have been reported to be potent bioactive compounds [12–13]. Additionally the terminal double bond of undecenoic acid provides a reactive group for further derivatization for producing potential functional derivatives. The synthetic route followed for the synthesis of the phenolipids is shown in Scheme 1. Initially, undecenoic acid was treated with sulfuric acid in methanol to obtain methyl undecenoate (**1**) in quantitative yield. Next, ester **1** was treated with 1,1′-azobis(cyclohexanecarbonitrile) (ABCN) and 2-mercaptoethylamine hydrochloride in dioxane/ethanol 70:30 (v:v) to obtain methyl 11-(2-aminoethylthio)undecanoate (**2**) in 89% yield. The structures of compounds **1** and **2** were in agreement with the reported literature data [14–15].

Finally, amine **2** was reacted with different phenolic acids in the presence of EDC·HCl and HOBt to give amides **3a–3e** with reproducible yields in the range of 84–86%.

### Anti-oxidant activity

The anti-oxidant activities of the prepared derivatives were studied by the well-established DPPH radical scavenging assay and also by studying the oxidation of linoleic acid using DSC. The DPPH radical is a commercially available stable free radical which is widely used to preliminarily determine the radical scavenging potential of compounds. The results for the ability of the prepared compounds to scavenge the DPPH radical are shown in Table 1 along with reference anti-oxidants α-tocopherol (α-TP) and *tert*-butylhydroquinone (TBHQ). As can be seen, all synthesized derivatives exhibit radical scavenging ability except the cinnamic acid derivative **3a**. This could be due to the absence of a phenolic hydroxy group which is responsible for the anti-oxidant activity of most of the natural phytochemicals. Among all compounds, **3c** was found to be the most efficient free radical scavenger which showed a value closest to the standard anti-oxidant, α-TP. Compounds **3d** and **3e** also showed free radical scavenging activity (FRSA) of 68% and 67%, respectively, whereas compound **3b** showed only moderate activity with 30% FRSA.

**Table 1:** DPPH radical scavenging activity of the synthesized 10-undecenoic acid methyl ester-based lipoconjugates.

Compound	FRSA (%) at 1.0 mM concentration

**3a**	–^a^
**3b**	30.23
**3c**	87.05
**3d**	67.68
**3e**	66.57
α-TP	90.23
TBHQ	92.34

^a^no activity.

In another study, the ability of the prepared derivatives in inhibiting the oxidation of linoleic acid was studied by differential scanning calorimetry (DSC). DSC is a sensitive technique and has been used for investigating the thermotropic properties of several compounds including biological macromolecules, drugs and lipid-based materials for their stability and other characteristics [16]. In the present study, pure linoleic acid and linoleic acid containing the synthesized compounds were subjected to DSC analysis. The results of the assay are shown in Table 2 and α-TP and TBHQ were included as standard anti-oxidants.

**Table 2:** DSC study of the synthesized 10-undecenoic acid methyl ester–based lipoconjugates **3a–e**.

Compound^a^	OIT^b^ (°C)

LA + **3a**	116
LA + **3b**	130
LA + **3c**	136
LA + **3d**	141
LA + **3e**	142
LA + α-TP	130
LA +TBHQ	126
LA	116

^a^LA: linoleic acid, ^b^OIT: oxidative induction temperature.

Pure linoleic acid showed an oxidative induction temperature (OIT) of 116 °C which was found to increase when anti-oxidants were added. It can be observed that the prepared derivatives had a positive influence on the oxidation of linoleic acid except for derivative **3a** which did not show any anti-oxidant activity (see Supporting Information File 1, Figure S20 for DSC curves). All other derivatives were found to exhibit very good protective activity against oxidation of linoleic acid and the OITs were found to be similar or in case of compounds **3c**, **3d** and **3e** even higher compared to the reference anti-oxidants TBHQ and α-TP. The OIT for TBHQ and α-TP were observed to be 126 and 130 °C, respectively, whereas compound **3b** showed an OIT of 130 °C. As the anti-oxidant activity has been reported [17] to depend on several factors such as the medium of an assay, number and position of phenolic hydroxy groups, etc. the differences in the anti-oxidant potentials of the prepared phenolipids could be due to different media used for the assays; the DPPH assay is conducted in a polar medium but the linoleic acid oxidation study is conducted in a non-polar environment.

### Cytotoxic activity

As there were studies reported on the cytotoxicity of phenolic lipids, we have further screened the prepared compounds for their anticancer activity [18]. The anticancer activity of compounds **3a**–**e** was tested against five cell lines along with doxorubicin as positive control and all of them showed moderate to good anticancer effects. The results are collected in Table 3. The compounds whose IC_50_ values are observed to be lower and closer to the reference drug are considered as having good anticancer potential.

**Table 3:** Anticancer activity of 10-undecenoic acid methyl ester–based lipoconjugates.^a^

Entry	Compound	IC_50_ values (μM)				

		MDA-MB-231	SKOV3	MCF7	DU145	HepG2
		
1	**3a**	21.2 ± 0.31	99.2 ± 0.79	17.2 ± 0.23	25.4 ± 0.31	38.2 ± 0.42
2	**3b**	14.5 ± 0.26	31.5 ± 0.41	39.2 ± 0.45	81.6 ± 0.77	58.3 ± 0.61
3	**3c**	12.0 ± 0.28	38.9 ± 0.37	10.55 ± 0.27	13.0 ± 0.26	67.4 ± 0.59
4	**3d**	29.0 ± 0.36	32.2 ± 0.32	28.8 ± 0.36	56.7 ± 0.62	93.9 ± 0.85
5	**3e**	12.5 ± 0.25	38.3 ± 0.40	13.9 ± 0.22	28.8 ± 0.39	141.4 ± 0.98
	doxorubicin	0.8 ± 0.14	0.7 ± 0.16	0.8 ± 0.12	0.8 ± 0.15	0.7 ± 0.14

^a^Cell lines: MDA-MB-231, breast cancer (ATCC^®^ HTB-26™); SKOV3, ovarian cancer (ATCC^®^ HTB-77™); MCF7, breast cancer (ATCC^®^ HTB-22™); DU 145, prostate cancer (ATCC^®^ HTB-81™); HepG2, liver hepatocellular carcinoma (ATCC^®^ HB-8065™).

Specifically compounds **3c**, **3b** and **3e** were found to show promising anticancer activity according to their IC_50_ values, whereas **3d** and **3a** exhibited only moderate activity. Among all tested derivatives, compound **3c** was found to exhibit best anticancer activity against MCF7, DU145 and MDA-MB-231 cell lines with IC_50_ values of 10.55, 13.0 and 12.0 µM, respectively. It was found that the anticancer activity against some cell lines was much better compared to our previous reports on phenolic lipids [19]. However, all prepared derivatives were observed to exhibit lower anticancer activity when compared to the reference drug doxorubicin which showed IC_50_ values in the range of 0.7 to 0.8 µM against the tested cell lines.

## Conclusion

In conclusion, the synthesis of five novel methyl 10-undecenoate-based lipoconjugates of phenolic acids is reported. The synthetic route was simple with product yields in the range of 84–86% over three steps. The lipid part, methyl 10-undecenoate was subjected to a thiol–ene reaction with cysteamine hydrochloride and the resulting intermediate was conjugated with the phenolic acid via amidation. The evaluation of the five novel phenolic lipids as anti-oxidants was studied using the DPPH radical scavenging assay and DSC studies where some compounds showed excellent anti-oxidant activity. Finally the compounds were further screened for anticancer activity where a few derivatives showed interesting activity.

## Experimental

### Materials

10-Undecenoic acid, 1,1’-azobis(cyclohexanecarbonitrile) (ABCN), hydroxybenzotriazole (HOBt) and 1-ethyl-3-(3’-dimethylaminopropyl)carbodiimide hydrochloride (EDC·HCl), cysteamine hydrochloride, cinnamic acid, sinapic acid, ferulic acid, *p*-coumaric acid, caffeic acid, α-tocopherol (α-TP), *tert*-butylhydroquinone (TBHQ), linoleic acid (LA) and 2,2-diphenyl-1-picrylhydrazyl (DPPH) radical were purchased from Sigma–Aldrich (St.Louis, USA), and pre-coated TLC plates (silica gel 60 F254) were purchased from Merck (Darmstadt, Germany). All solvents were purchased from Sd Fine Chemicals (Mumbai, India) and were of the highest grade of purity available.

### Instruments

^1^H and ^13^C NMR spectra were recorded on a Bruker Avance operating at 700/500 MHz and 175/125 MHz. The NMR spectra were referenced to δ 7.26 ppm and δ 77.0 ppm in CDCl_3_ solvent for ^1^H and ^13^C, respectively. Coupling constants (*J*) patterns in the ^1^H NMR spectra are given as follows: s = singlet, d = doublet, t = triplet, q = quartet, m = multiplet protons. Mass spectra were recorded using electron spray ionization (ESI) on a Waters e2695 Separators module (Waters, Milford, MA, USA) mass spectrometer. FTIR spectra were recorded in chloroform on a Perkin–Elmer Fourier Transform (FTIR) spectrum BX instrument (Model: Spectrum BX; Connecticut, USA). HRMS spectra were obtained from an Exactive Orbitrap mass spectrometer (Thermo Scientific, Waltham, MA, USA). Melting points of lipoconjugates of phenolic molecules were determined with a capillary tube melting point apparatus. Gas chromatography (GC) was performed on an Agilent 6890N gas chromatograph (Delaware, USA) equipped with a flame ionization detector using a HP-1 capillary column (30 m × 0.25 mm × 0.25 μm). The injector and detector temperatures were set at 280 and 300 °C, respectively. The oven temperature was programmed at 150 °C for 2 min and then increased to 300 °C at 10 °C/min and final temperature hold for 20 min. The carrier gas used was nitrogen at a flow rate of 1.0 mL/min.

### Methods

**Synthesis of methyl undec-10-enoate (1):** 10-Undecenoic acid (10 g, 54.34 mmol), was added to methanol (17.6 mL) and sulfuric acid (0.1 mL, 2 wt % 10-undecenoic acid) and stirred at refluxing temperature of methanol for 6 h. After completion of the reaction as shown by TLC (hexane/ethyl acetate 80:20, v/v), excess methanol was removed under reduced pressure and the product was diluted with ethyl acetate (30 mL), washed with 5% aqueous NaHCO_3_ solution (3 × 30 mL), and dried over anhydrous Na_2_SO_4_. The organic solvent was removed under reduced pressure to afford crude methyl ester of 10-undecenoic acid. The product was purified by column chromatography with basic alumina and hexane as the eluent to get 99% pure methyl undec-10-enoate (**1**) as indicated by GC. The product was analyzed by ^1^H NMR, ^13^C NMR, ESIMS, and FTIR and the structure was confirmed by comparing the data with those reported in the literature [14].

**Synthesis of methyl 11-(2-aminoethylthio) undecanoate (2):** For the synthesis of compound **2**, a reported protocol was followed with slight modifications [20]. Briefly, methyl undecenoate (**1**, 6 g, 30.3 mmol) and ABCN (0.18 g, 3 wt % of **1**) were dissolved in 40 mL chloroform. Then, 2-mercaptoethylamine hydrochloride (6.8 g, 60 mmol) and 40 mL of 1,4-dioxane/ethanol (70:30; v/v) were added and the mixture was stirred at 85 °C for 48 h. The progress of the reaction was monitored by TLC (hexane/ethyl acetate 80:20, v/v). After maximum conversion, the reaction mixture was extracted with dichloromethane (2 × 40 mL) and the combined organic phases were washed with saturated K_2_CO_3_, brine and finally with water and dried over anhydrous Na_2_SO_4_. This crude product mixture was concentrated and purified by column chromatography with hexane/ethyl acetate (92:8, v/v) to obtain pure methyl 11-(2-aminoethylthio)undecanoate (**2**) in 89% yield (7.41 g). The purified product was characterized by ^1^H and ^13^C NMR, IR and ESIMS spectral studies and the structure was confirmed by comparing the data with those reported in the literature [15].

**Synthesis of methyl 11-((2-(cinnamamido)ethyl)sulfanyl)undecanoate (3a):** The amidation reaction was performed following a reported protocol with slight modifications [21]. Briefly, compound **2** (0.58 g, 2.1 mmol) and cinnamic acid (0.4 g, 3.1 mmol) were dissolved in dichloromethane (30 mL) and the mixture was stirred at 0–5 °C under a nitrogen atmosphere. EDC·HCl (0.4 g, 2.52 mmol) and HOBt (0.3 g, 3.1 mmol) were added and the contents were stirred at 0–5 °C for 10 min. After the addition, the mixture was stirred for 12 h at rt under a nitrogen atmosphere and the progress of reaction was monitored by TLC using the solvent system chloroform/methanol (80:20, v/v). After maximum conversion, the reaction mixture was extracted with dichloromethane, washed with water and dried over anhydrous Na_2_SO_4_ and concentrated to obtain the crude product. The crude product was purified by column chromatography (chloroform/methanol 90:10, v/v) to obtain the thioamide of cinnamic acid in 86% yield (0.73 g). The product was characterized by ^1^H and ^13^C NMR, IR, ESIMS and HRMS spectral studies. Mp 55–56 °C; ^1^H NMR (500 MHz, CDCl_3_) δ 7.64 (d, *J* = 15.6 Hz, 1H), 7.54–7.46 (m, 5H), 6.24 (d, *J* = 15.6 Hz, 1H), 3.66 (s, 3H), 3.56 (q, 2H), 2.73 (t, 2H), 2.58 (t, 2H), 2.30 (t, 2H), 1.24–1.62 (m, 12H, CH_2_); ^13^C NMR (75 MHz, CDCl_3_) δ 174.37 (-*C*(O)-OCH_3_), 165.50 (-NH-*C*(O)-), 141.25 (-NH-C(O)-CH=*C*H-),134.81–127.83, 120.52 (-NH-C(O)-*C*H=CH-), 51.47 (-C(O)-O*C*H_3_-), 38.49 (-*C*H_2_-NH-), 29.44 (-S-*C*H_2_-), 29.36 (-*C*H_2_-S-), 29.22 (-*C*H_2_-CH_2_-S-), 29.19–24.96 (-CH_2_-CH_2_-); IR (cm^−1^, KBr): 2853, 2853, 1720, 1654, 1599, 1527, 1441, 1365; ESIMS (*m*/*z*): 406 [M + H]^+^, 428 [M + Na]^+^; HRMS (*m*/*z*): [M + H]^+^ calcd for C_23_H_36_O_3_NS, 406.24104; found, 406.24077.

**Synthesis of methyl 11-((2-((*E*)-3-(4-hydroxyphenyl)acrylamido)ethyl)sulfanyl)undecanoate (3b):** Similarly, methyl 11-((2-((*E*)-3-(4-hydroxyphenyl)acrylamido)ethyl)sulfanyl)undecanoate (**3b**) was prepared from **2** (0.6 g, 2.1 mmol) and coumaric acid (0.5 g, 3.2 mmol) in 85% yield (0.78 g) and the product was characterized by ^1^H and ^13^C NMR, IR, ESIMS and HRMS spectral studies. Mp 64–65 °C; ^1^H NMR (500 MHz, CDCl_3_) δ 7.57 (d, *J* = 15.6 Hz, 1H), 7.39 (d, *J* = 8.6 Hz, 1H), 6.85 (d, *J* = 8.6 Hz, 1H), 6.26 (d, *J* = 15.6 Hz, 1H), 3.67 (s, 3H), 3.58 (q, 2H), 2.73 (t, 2H), 2.58 (t, 2H), 2.30 (t, 2H), 1.24–1.62 (m, 12H, CH_2_); ^13^C NMR (75 MHz, CDCl_3_) δ 174.71 (-*C*(O)-OCH_3_), 166. 84 (-NH-*C*(O)-), 158.33, 141.51 (-NH-C(O)-CH=*C*H-),129.64, 128.99, 117.32 (-NH-C(O)-*C*H=CH-), 115.98, 51.58 (-C(O)-O*C*H_3_-), 38.63 (-*C*H_2_-NH-), 34.16 (-S-*C*H_2_-), 31.89 (-*C*H_2_-S-), 31.84 (-*C*H_2_-CH_2_-S-), 29.69–24.96 (-CH_2_-CH_2_-); IR (cm^−1^, KBr): 3409, 2923, 2853, 1729, 1652, 1595, 1519, 1452, 1373; ESIMS (*m*/*z*): 422 [M + H]^+^, 444 [M + Na]^+^; HRMS (*m*/*z*): [M + H]^+^ calcd for C_26_H_36_O_4_NS, 422.23596; found, 422.23491.

**Synthesis of methyl 11-((2-((*E*)-3-(3,4-dihydroxyphenyl)acrylamido)ethyl)sulfanyl)undecanoate (3c):** Similarly, methyl 11-((2-((*E*)-3-(3,4-dihydroxyphenyl)acrylamido)ethyl)sulfanyl)undecanoate was prepared from **2** (0.6 g, 2.1 mmol) and caffeic acid (0.5 g, 3.2 mmol) in 85% yield (0.81 g) and the product was characterized by ^1^H and ^13^C NMR, IR, ESIMS and HRMS spectral studies. ^1^H NMR (500 MHz, CDCl_3_) δ 7.55 (d, *J* = 15.6 Hz, 1H), 7.12 (d, *J* = 8.6 Hz, 1H), 6.95 (s, 1H) 6.87 (d, *J* = 8.6 Hz, 1H), 6.24 (d, *J* = 15.6 Hz, 1H), 3.68 (s, 3H), 3.56 (q, 2H), 2.73 (t, 2H), 2.58 (t, 2H), 2.30 (t, 2H), 1.24–1.62 (m, 12H, CH_2_); ^13^C NMR (75 MHz, CDCl_3_) δ 174.74 (-*C*(O)-OCH_3_), 167. 38 (-NH-*C*(O)-), 146.87, 144.53, 142.53 (-NH-C(O)-CH=*C*H-), 127.07, 121.33, 117.12 (-NH-C(O)-*C*H=CH-), 115.46, 114.71, 51.60 (-C(O)-O*C*H_3_-), 39.05 (-*C*H_2_-NH-), 38.88 (-S-*C*H_2_-), 38.76 (-*C*H_2_-S-), 34.16 (-*C*H_2_-CH_2_-S-), 29.59–24.96 (-CH_2_-CH_2_-); IR (cm^−1^, KBr): 3359, 2953, 2854, 1721, 1654, 1599, 1527, 1441, 1365; ESIMS (*m*/*z*): 438 [M + H]^+^, 460 [M + Na]^+^; HRMS (*m*/*z*): [M + H]^+^ calcd for C_23_H_36_O_5_NS, 438.23087; found, 438.23023.

**Synthesis of methyl 11-((2-((*E*)-3-(4-hydroxy-3-methoxyphenyl)acrylamido)ethyl)sulfanyl)undecanoate (3d):** Similarly, methyl 11-((2-((*E*)-3-(4-hydroxy-3-methoxyphenyl)acrylamido)ethyl)sulfanyl)undecanoate was prepared from **2** (0.6 g, 2.1 mmol) and ferulic acid (0.6 g, 3.2 mmol) in 84% yield (0.82 g) and the product was characterized by ^1^H and ^13^C NMR, IR, ESIMS and HRMS spectral studies. ^1^H NMR (500 MHz, CDCl_3_) δ 7.55 (d, *J* = 15.5 Hz, 1H), 7.06 (dd, *J* = 8.2, 1.5 Hz, 1H), 7.00 (d, *J* = 1.6 Hz, 1H), 6.91 (d, *J* = 8.2 Hz, 1H), 6.27 (d, *J* = 15.5 Hz, 1H), 3.92 (s, 3H), 3.67 (s, 3H), 3.58 (q, 2H), 2.73 (t, 2H), 2.58 (t, 2H), 2.30 (t, 2H), 1.24–1.62 (m, 12H, CH_2_); ^13^C NMR (75 MHz, CDCl_3_) δ 174.36 (-*C*(O)-OCH_3_), 166. 21 (-NH-*C*(O)-), 147.48, 146.76, 141.26 (-NH-C(O)-CH=*C*H-), 127.33, 122.22, 118.06 (-NH-C(O)-*C*H=CH-), 114.76, 109.64, 55.95 (-OCH_3_-), 51.45 (-C(O)-O*C*H_3_-), 38.42 (-*C*H_2_-NH-), 34.11 (-S-*C*H_2_-), 31.97 (-*C*H_2_-S), 31.74 (-*C*H_2_-CH_2_-S-), 29.47–24.95 (-CH_2_-CH_2_-); IR (cm^−1^, KBr): 3375, 2926, 2853, 1730, 1656, 1596, 1516, 1433, 1273; ESIMS (*m*/*z*): 452 [M + H]^+^, 474 [M + Na]^+^; HRMS (*m*/*z*): [M + H]^+^ calcd for C_24_H_38_O_5_NS, 452.24652; found, 452.24475.

**Synthesis of methyl-11-((2-((*E*)-3-(4-hydroxy-3,5-dimethoxyphenyl)acrylamido)ethyl)sulfanyl)undecanoate (3e):** Similarly, methyl 11-((2-((*E*)-3-(4-hydroxy-3,5-dimethoxyphenyl)acrylamido)ethyl)sulfanyl)undecanoate was prepared from **2** (0.6 g, 2.1 mmol) and sinapic acid (0.7 g, 3.2 mmol) in 85% yield (0.89 g) and the product was characterized by ^1^H and ^13^C NMR, IR, ESIMS and HRMS spectral studies. Mp 69–70 °C; ^1^H NMR (500 MHz, CDCl_3_) δ 7.53 (d, *J* = 15.5 Hz, 1H), 6.76 (s, *J* = 8.2, 1.5 Hz, 2H), 6.28 (d, *J* = 15.5 Hz, 1H), 3.93 (s, 6H), 3.67 (s, 3H), 3.58 (q, 2H), 2.73 (t, 2H), 2.58 (t, 2H), 2.30 (t, 2H), 1.24–1.62 (m, 12H, CH_2_); ^13^C NMR (75 MHz, CDCl_3_) δ 174.37 (-*C*(O)-OCH_3_), 166.07 (-NH-*C*(O)-), 147.22, 141.43 (-NH-C(O)-CH=*C*H-),136.64, 126.29, 118.44 (-NH-C(O)-*C*H=CH-), 104.82, 56.34 (-OCH_3_-), 51.48 (-C(O)-O*C*H_3_-), 38.39 (-*C*H_2_-NH-), 34.11 (-S-*C*H_2_-), 31.96 (-*C*H_2_-S-), 31.69 (-*C*H_2_-CH_2_-S-), 29.65–24.95 (-CH_2_-CH_2_-); IR (cm^−1^, KBr): 3371, 2926, 2852, 1730, 1658, 1612, 1514, 1455 1285; ESIMS (*m*/*z*): 482 [M + H]^+^, 504 [M + Na]^+^; HRMS (*m*/*z*): [M + H]^+^ calcd for C_25_H_40_O_6_NS, 482.25709; found, 482.25532.

### Anti-oxidant activity

#### DPPH radical scavenging assay

The anti-oxidant activity was determined by the radical scavenging ability using the stable DPPH radical method as reported [22]. Briefly, 200 µL of a methanolic solution of the synthesized phenolic lipoconjugates (1 mM concentrations) were added to 2 mL of a methanolic solution of the DPPH radical (1 mM concentration) and the total volume was made up to 3 mL with methanol. After 40 min of standing, the absorbance of the mixture was measured at 517 nm against methanol as blank sample. TBHQ and α-TP (1 mM concentration) were used as a positive control. The radical-scavenging activities (%) of the tested samples were evaluated by comparison with the control (2 mL DPPH radical solution and 1 mL methanol). Each sample was measured in triplicate and averaged. The free-radical scavenging activity (FRSA) was calculated using the following formula: FRSA = [(*A*c − *A*s)/*A*c] × 100 where *A*c is the absorbance of the control and *A*s is the absorbance of the tested sample after 40 min.

#### DSC measurements

The anti-oxidant activity was also evaluated by differential scanning calorimetry using pure linoleic acid as a lipid model system [23–24]. All studied anti-oxidants were dissolved in methanol to prepare 1 mM solutions. Samples of linoleic acid (2.5–3.0 mg) were placed in standard aluminum pans and spiked with 10 µL of the anti-oxidant solution. A blank run of linoleic acid, spiked with 10 µL of methanol was also carried out simultaneously to find the oxidative induction temperature (OIT) of linoleic acid. OIT is determined from the first exothermal peak of the plot of heat flow (mW/g) vs temperature. All measurements for each compound were run in triplicate and the results were averaged.

#### Cytotoxicity test (MTT assay)

The cytotoxicity assay (MTT) was evaluated for all test compounds as described in our earlier work [25]. Five different cancer cell lines viz., MDA-MB-231, breast cancer (ATCC^®^ HTB-26™); SKOV3, ovarian cancer (ATCC^®^ HTB-77™); MCF7, breast cancer (ATCC^®^ HTB-22™); DU 145, prostate cancer (ATCC^®^ HTB-81™); HepG2, liver hepatocellular carcinoma (ATCC^®^ HB-8065™) were obtained from the ATCC (Bethesda, MD, USA) and maintained in DMEM supplemented with 10% FBS, 2 mM L-glutamine, 100 U/mL penicillin, and 100 μg/mL streptomycin at 37 °C in a 5% CO_2_ incubator. After seeding of cells in 96 well culture plates, they were allowed to attach properly. Test compounds of different concentrations ranging from 1 to 50 µM were added in triplicates and incubated for 24 h. The cells were then incubated with MTT (0.5 mg/mL) for 3 h and, to dissolve the insoluble formazan crystals, 100 µL DMSO was added to each well. Finally the absorbance of the plates was measured using a Synergy H1 multi-mode plate reader (USA). Doxorubicin was used as the positive control for comparison.

## Supporting Information

File 1 Copies of ^1^H NMR, ^13^C NMR, HRMS and DSC spectra.
