# Supplementary material for: Synthesis and evaluation of anti-oxidant and cytotoxic activities of novel 10-undecenoic acid methyl ester based lipoconjugates of phenolic acids
Source: Beilstein J Org Chem. 2017 Jan 4;13:26–32. doi: 10.3762/bjoc.13.4 (PMC5238565; doi:10.3762/bjoc.13.4)
Supplement: File 1 — Copies of 1H NMR, 13C NMR, HRMS and DSC spectra. [file Beilstein_J_Org_Chem-13-26-s001.pdf]

Supporting Information

for

Synthesis and evaluation of anti-oxidant and cytotoxic activities of novel 10-undecenoic acid methyl ester based lipoconjugates of phenolic acids

Naganna Narra[1,2], Shiva Shanker Kaki*[§1,2], Rachapudi Badari Narayana Prasad[1,2], Sunil Misra[2,3], Koude Dhevendar[2,3], Venkateshwarlu Kontham[1,2], and Padmaja V. Korlipara*[§1,2]

Address: [1]Centre for Lipid Research, CSIR-Indian Institute of Chemical Technology, Uppal Road, Hyderabad 500007, India, [2]Academy of Scientific and Innovative Research, New Delhi, India and [3] Biology Division, CSIR-Indian Institute of Chemical Technology, Uppal Road, Hyderabad 500007, India

Email: Shiva Shanker Kaki* - shivashanker.kaki@iict.res.in; Padmaja V. Korlipara* - kvpadmaja@iict.res.in

*Corresponding author

[§]Tel/Fax +91-40-27193370

Copies of ^{1}H NMR, ^{13}C NMR, HRMS and DSC spectra

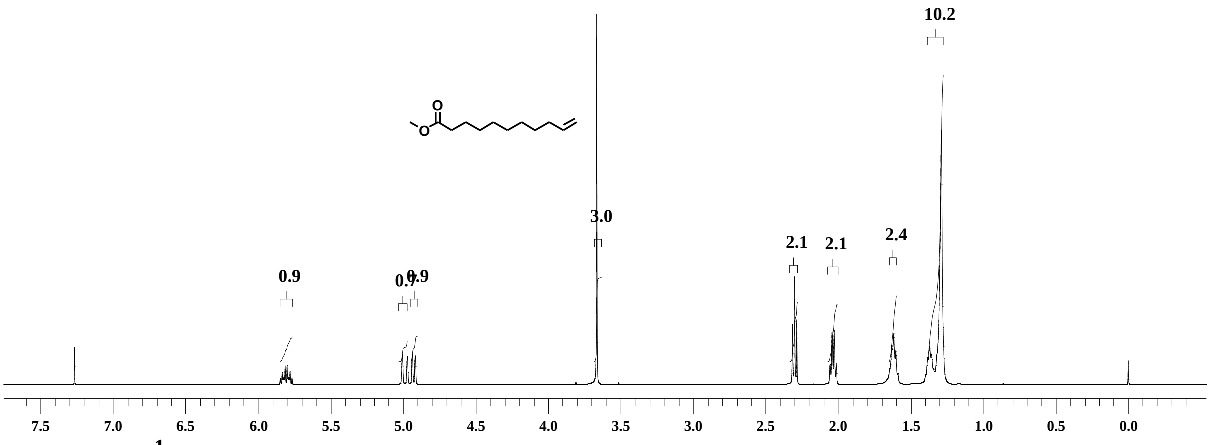

Figure S1: ^{1}H NMR spectrum of **1**

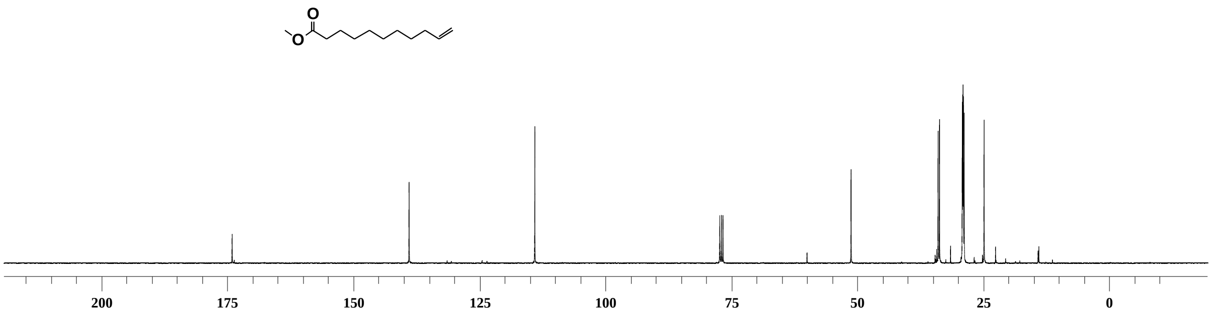

Figure S2: ^{13}C NMR spectrum of **1**

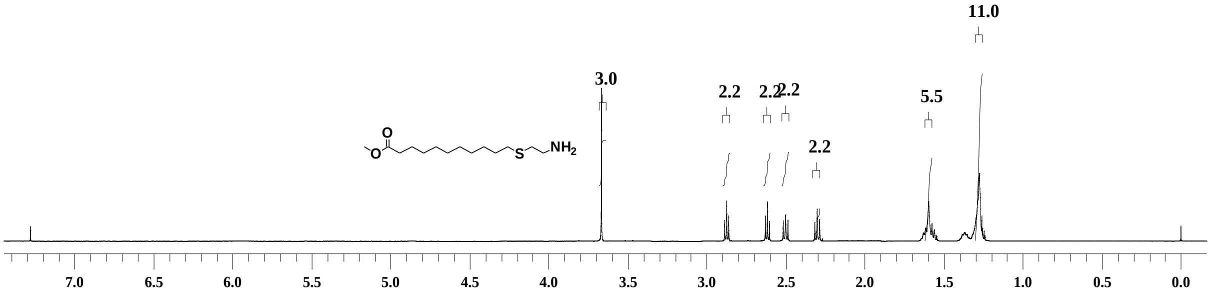

Figure S3: ^{1}H NMR spectrum of **2**

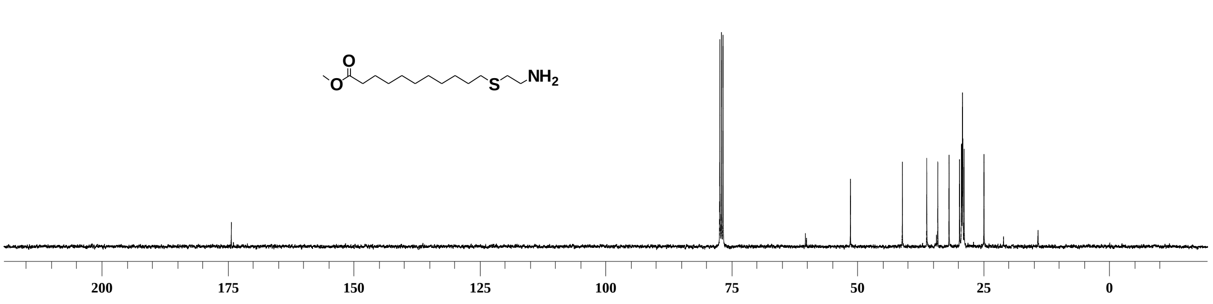

Figure S4: ^{13}C NMR spectrum of **2**

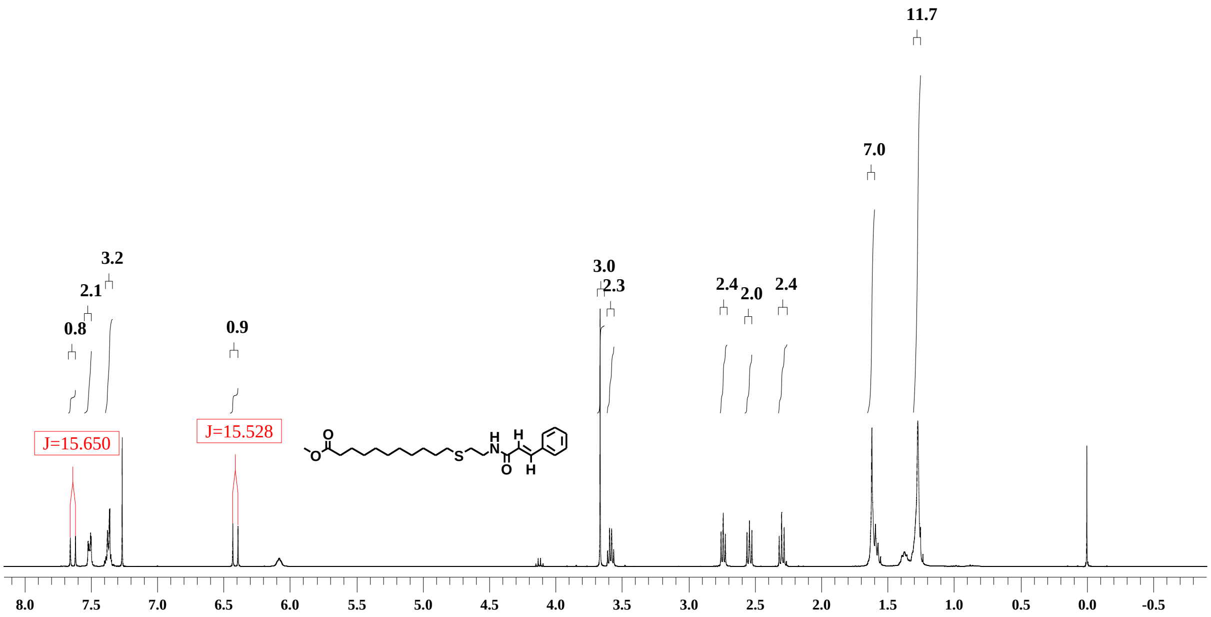

Figure S5: ^{1}H NMR spectrum of **3a**

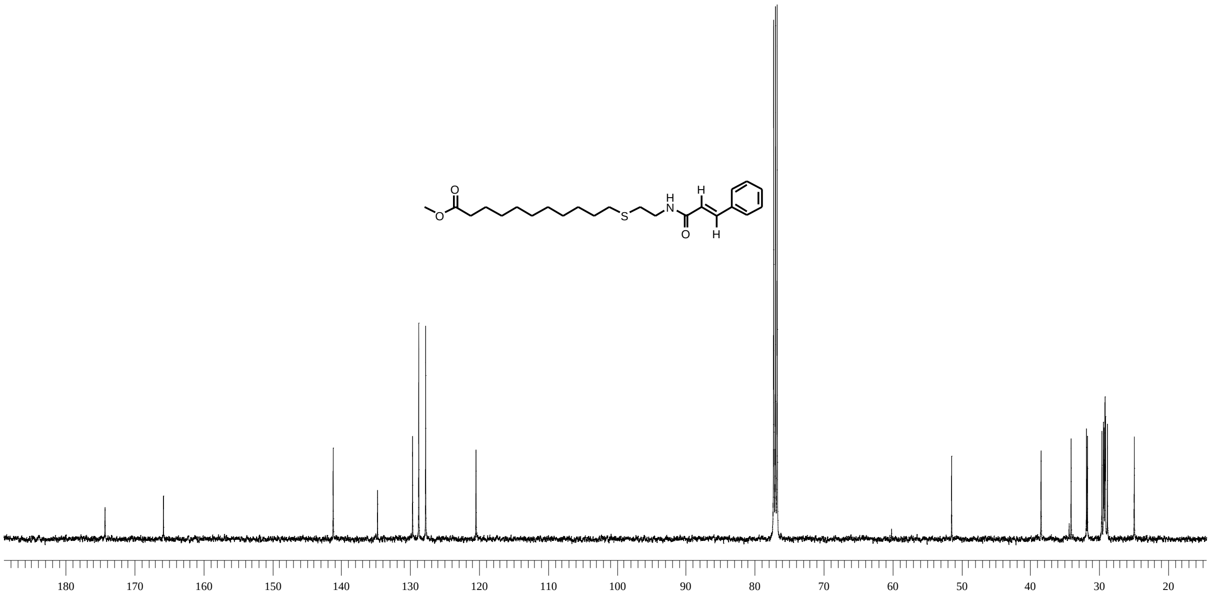

Figure S6: ^{13}C NMR spectrum of **3a**

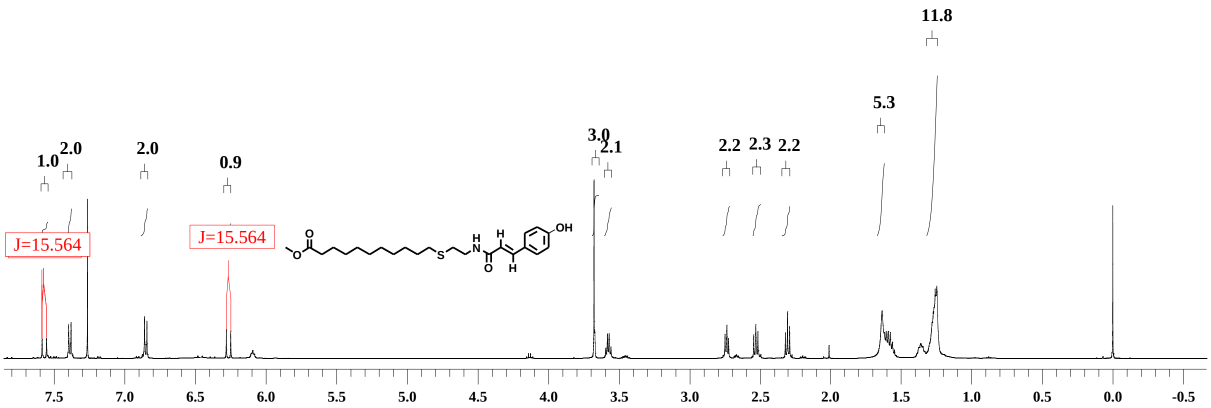

Figure S7: ^{1}H NMR spectrum of **3b**

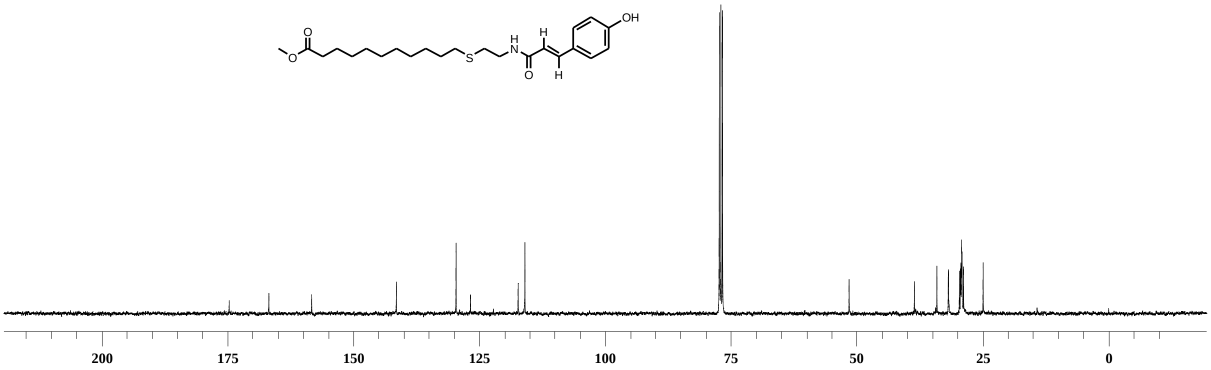

Figure S8: ^{13}C NMR spectrum of **3b**

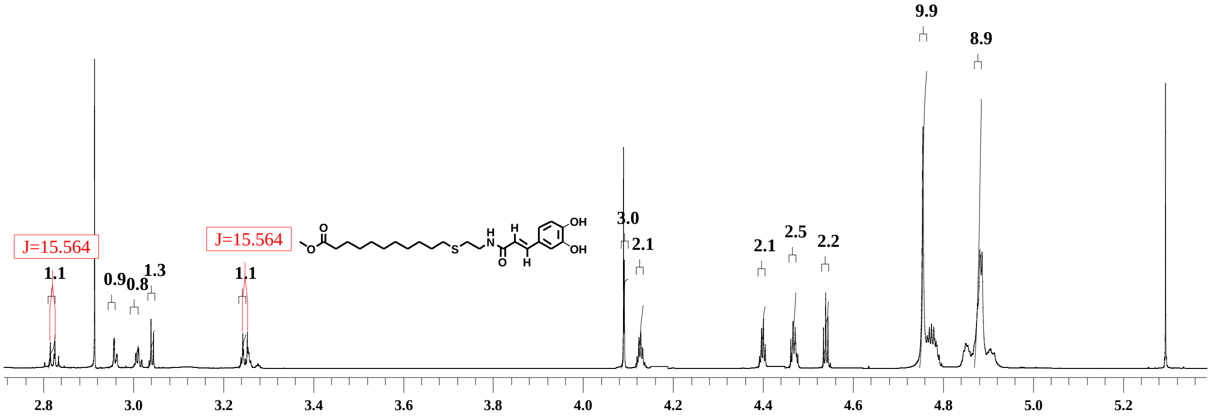

Figure S9: ^{1}H NMR spectrum of **3c**

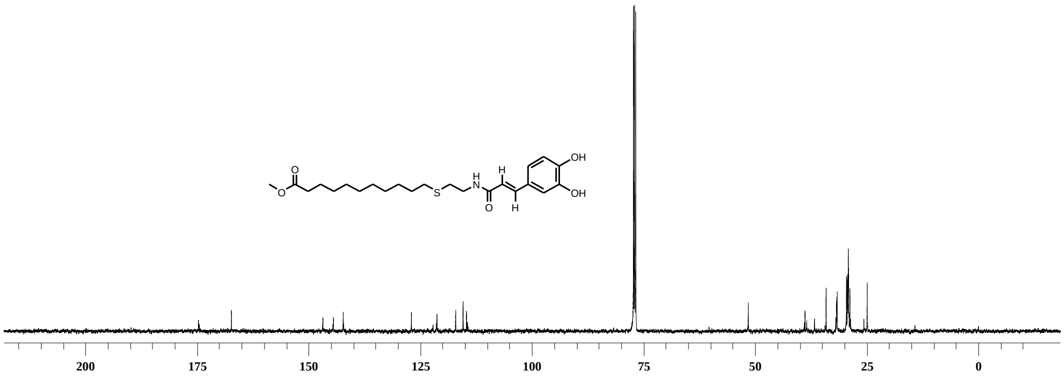

Figure S10: ^{13}C NMR spectrum of **3c**

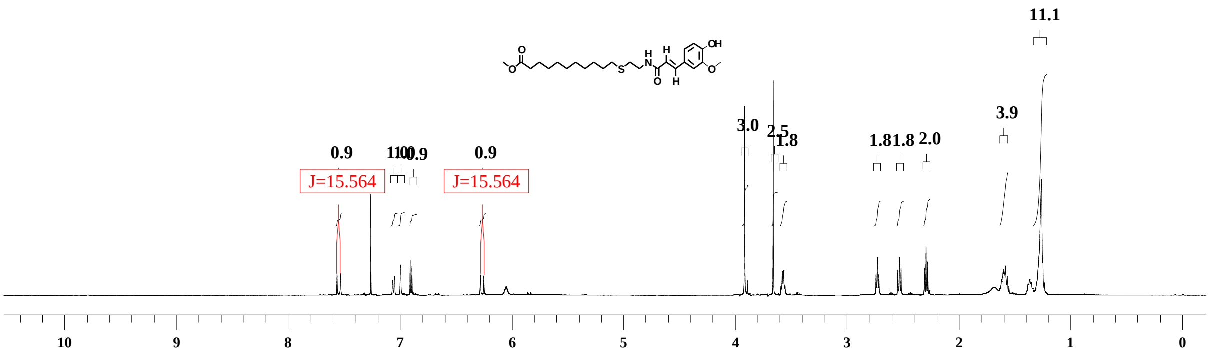

Figure S11: ^{1}H NMR spectrum of **3d**

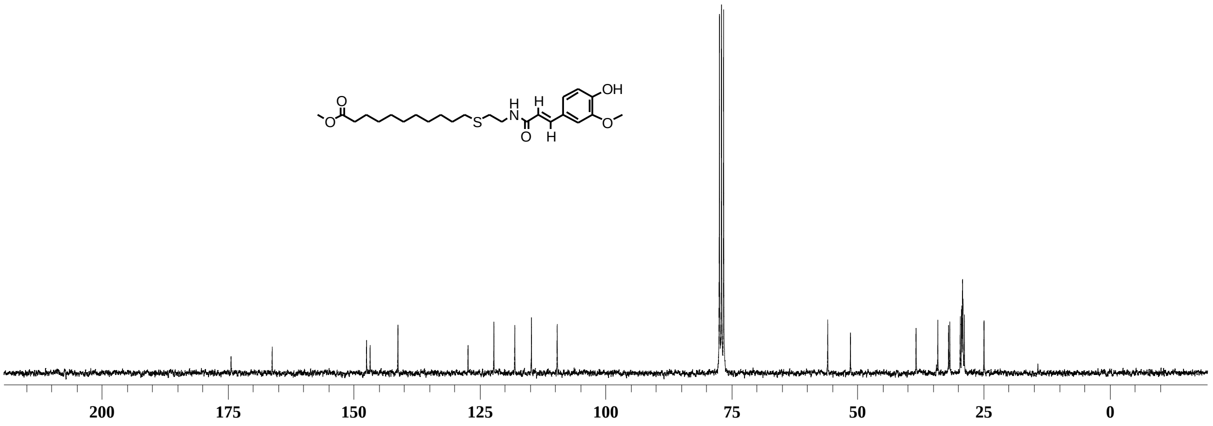

Figure S12: ^{13}C NMR spectrum of **3d**

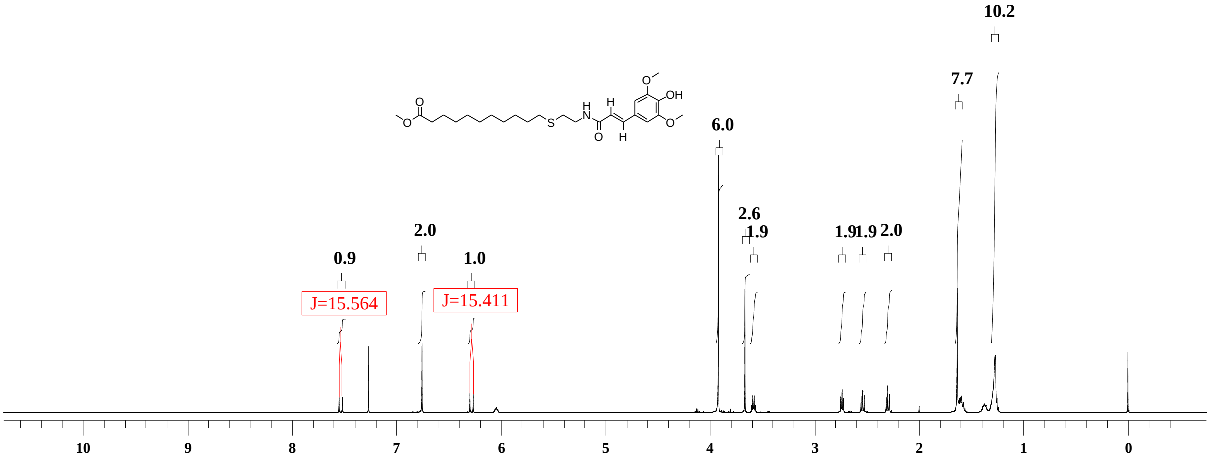

Figure S13: ^{1}H NMR spectrum of **3e**

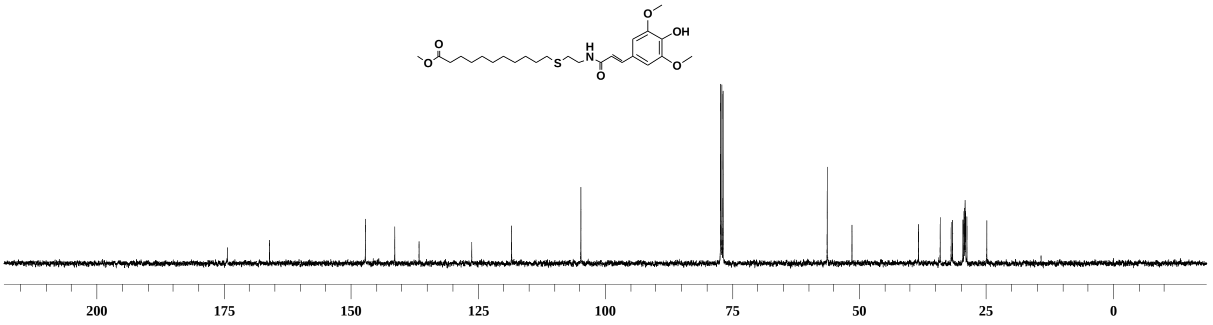

Figure S14: ^{13}C NMR spectrum of **3e**

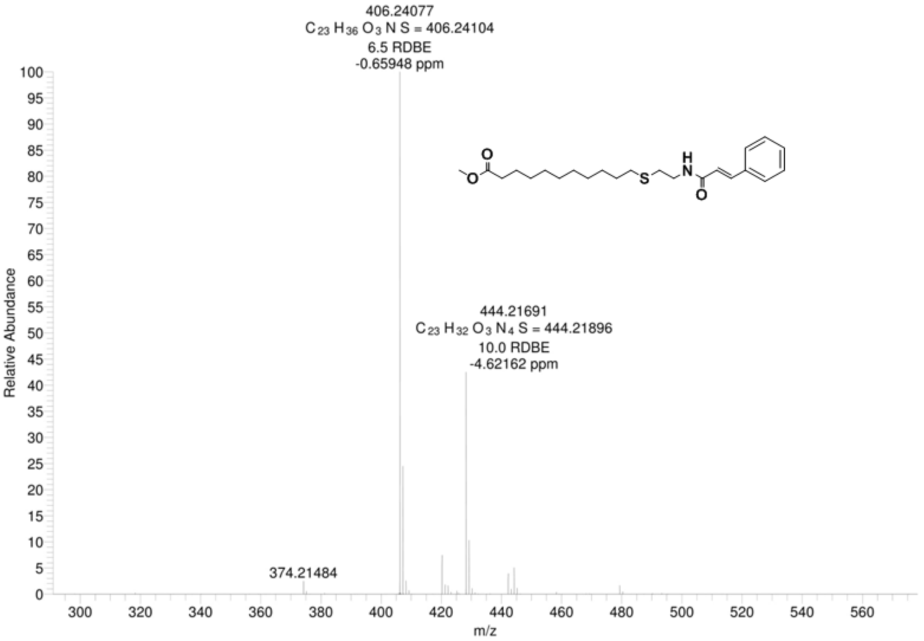

Figure S15: The HRMS spectrum of **3a**

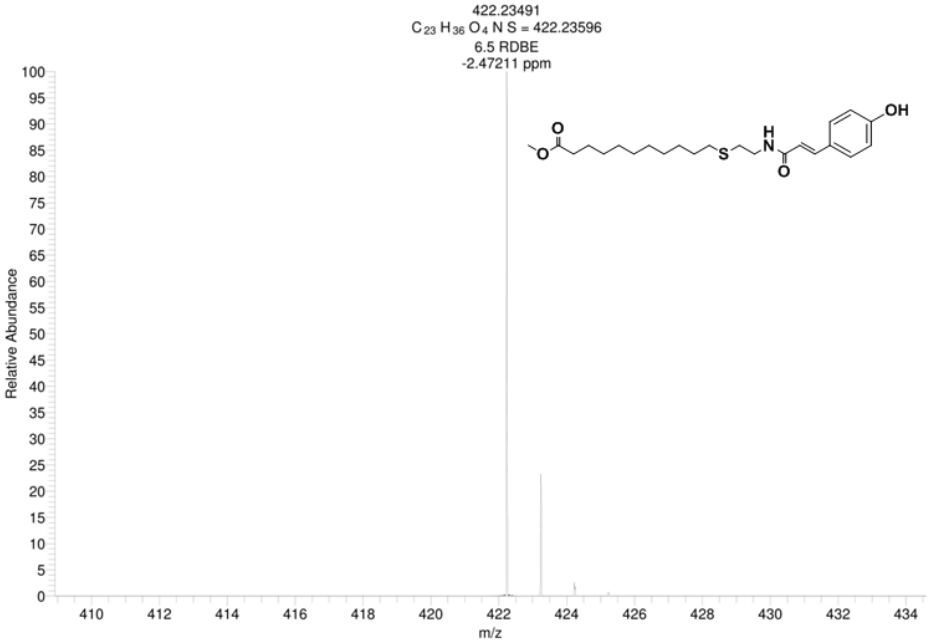

Figure S16: The HRMS spectrum of **3b**

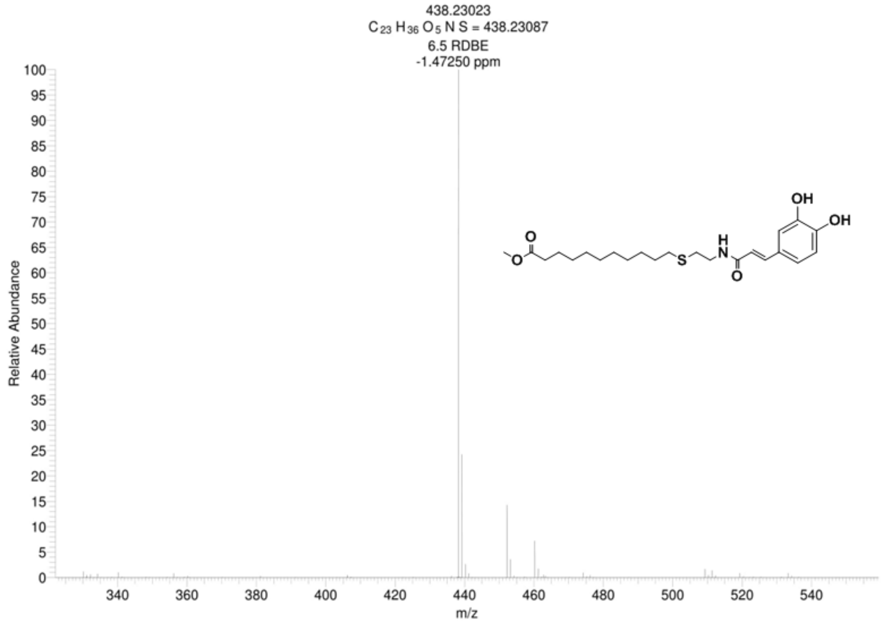

Figure S17: The HRMS spectrum of **3c**

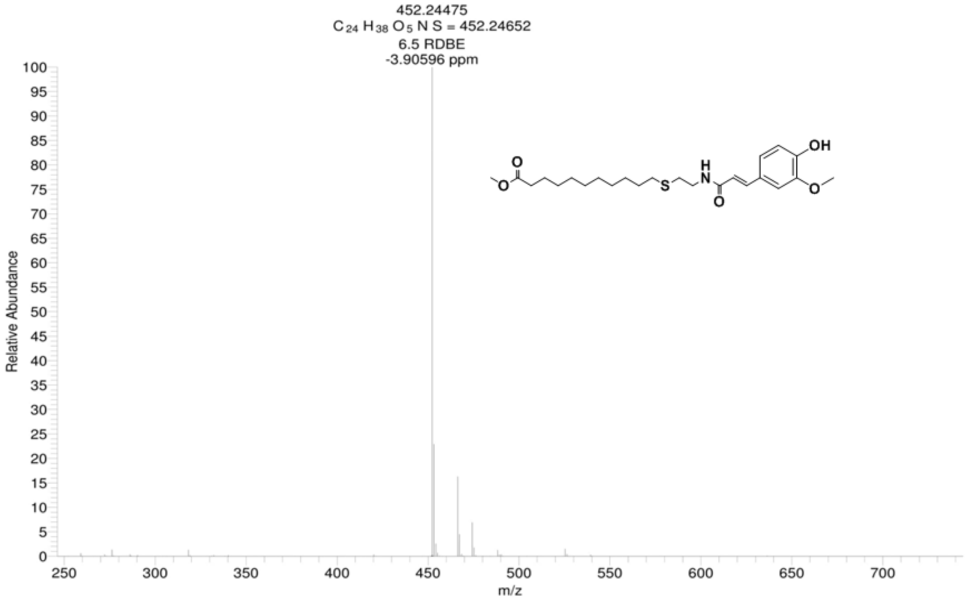

Figure S18: The HRMS spectrum of **3d**

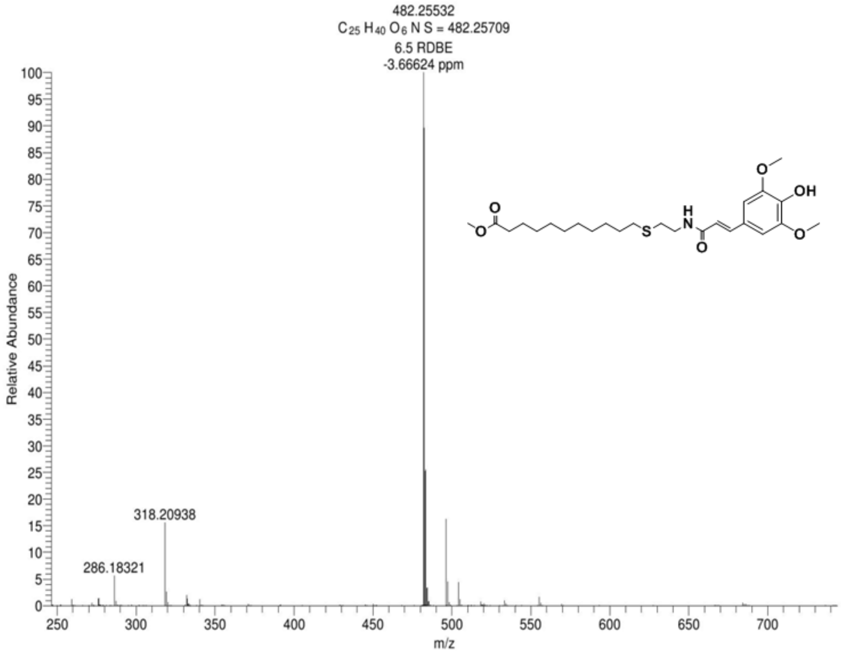

Figure S19: The HRMS spectrum of **3e**

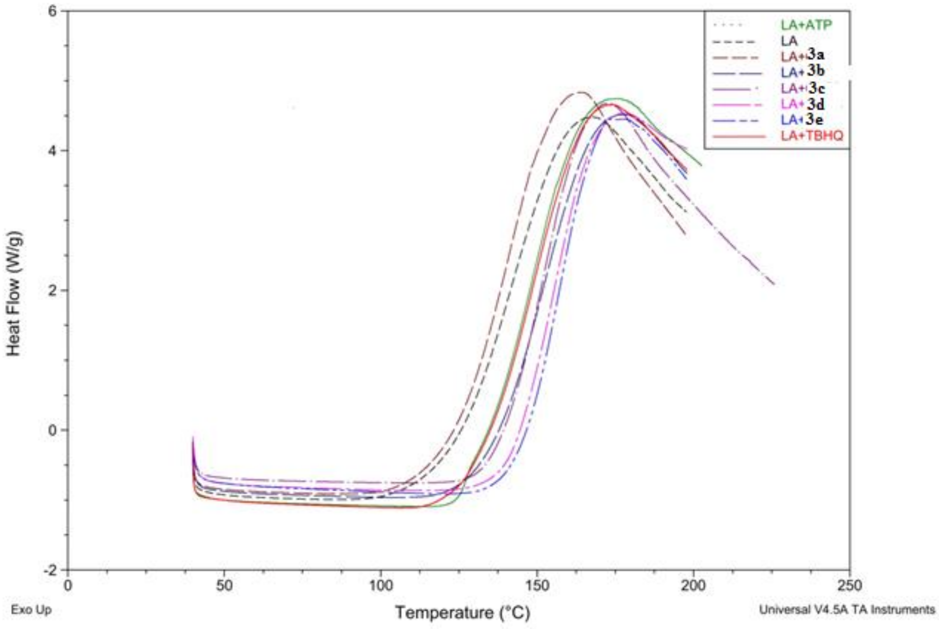

Figure S20: DSC curves of the synthesised 10-undecenoic acid –based lipoconjugates
